# Prebiotic Dietary Fiber and Gut Health: Comparing the in Vitro Fermentations of Beta-Glucan, Inulin and Xylooligosaccharide

**DOI:** 10.3390/nu9121361

**Published:** 2017-12-15

**Authors:** Justin L. Carlson, Jennifer M. Erickson, Julie M. Hess, Trevor J. Gould, Joanne L. Slavin

**Affiliations:** 1 Department of Food Science and Nutrition, University of Minnesota, 1334 Eckles Ave, St. Paul, MN 55108, USA; carl2814@umn.edu (J.L.C.); eric2472@umn.edu (J.M.E.); jmhess@umn.edu (J.M.H.); 2 Informatics Institute, University of Minnesota, 101 Pleasant St., Minneapolis, MN 55455, USA; goul0109@umn.edu

**Keywords:** prebiotic, microbiota, fermentation, dietary fiber, microbiome

## Abstract

Prebiotic dietary fiber supplements are commonly consumed to help meet fiber recommendations and improve gastrointestinal health by stimulating beneficial bacteria and the production of short-chain fatty acids (SCFAs), molecules beneficial to host health. The objective of this research project was to compare potential prebiotic effects and fermentability of five commonly consumed fibers using an in vitro fermentation system measuring changes in fecal microbiota, total gas production and formation of common SCFAs. Fecal donations were collected from three healthy volunteers. Materials analyzed included: pure beta-glucan, Oatwell (commercially available oat-bran containing 22% oat β-glucan), xylooligosaccharides (XOS), WholeFiber (dried chicory root containing inulin, pectin, and hemi/celluloses), and pure inulin. Oatwell had the highest production of propionate at 12 h (4.76 μmol/mL) compared to inulin, WholeFiber and XOS samples (*p* < 0.03). Oatwell’s effect was similar to those of the pure beta-glucan samples, both samples promoted the highest mean propionate production at 24 h. XOS resulted in a significant increase in the genus *Bifidobacterium* after 24 h of fermentation (0 h:0.67 OTUs (operational taxonomic unit); 24 h:5.22 OTUs; *p* = 0.038). Inulin and WholeFiber increased the beneficial genus *Collinsella*, consistent with findings in clinical studies. All analyzed compounds were fermentable and promoted the formation of beneficial SCFAs.

## 1. Introduction

Prebiotic definitions vary among different scientific and political arenas across the world [1]. Depending on the local definition, nearly all prebiotics can be classified as dietary fiber, but not all fibers are considered prebiotics [2]. The most recent definition describes a prebiotic as “a substrate that is selectively utilized by host microorganisms conferring a health benefit” [3]. Functional characteristics of prebiotics include the ability to: resist the low pH of the stomach, resist hydrolysis by mammalian enzymes, resist absorption in the upper gastrointestinal tract, the ability to be fermented by intestinal microbiota and selectively stimulate the growth and/or activity of intestinal bacteria associated with host health and overall well-being [4,5]. Inulin, beta-glucans, and xylooligosaccharides all provide health benefits to consumers that are related to the fermentation of these compounds in the distal gastrointestinal tract, and are also considered functional fibers with many other benefits [6]. As the definition of “prebiotic” broadens to include the overall impact from the metabolism from these compounds, the category of prebiotics will expand [7]. The importance of displaying direct health benefits due to bacterial fermentation is still the driving mechanism for all prebiotics.

As our awareness and understanding of the importance of the gut microbiome and gut microbiota increases, it is imperative for consumers to understand the key differences between different forms of prebiotics, and where they can be found in various foods and food products. XOS is an emerging prebiotic with well-displayed, consistent health benefits [8] and is composed of sugar oligomers composed of xylose units [9], found naturally in fruits, vegetables, milk, honey and bamboo shoots. XOS is commonly produced from xylan containing lignocellulosic materials through various chemical methods, direct enzymatic hydrolysis, or a combination of both treatments [10,11,12,13,14]. Inulin is a heterogeneous blend of fructose polymers (degree of polymerization, DP < 10) [15] which occurs naturally in thousands of plant species, including wheat, onion, bananas, garlic and chicory [16]. Beta-glucan is a polysaccharide composed of d-glucose monomers with beta-glycosidic linkages, present in either linear chains in grains, such as oat and barley (up to 7%), or in branched structures in fungi, yeast and certain bacteria [17]. These prebiotics, or prebiotic mixtures, each provide a unique carbon source for selective stimulation of different bacterial taxa and are important microbiota-shaping compounds.

Because no analytical method currently exists to measure the prebiotic capacity of foods in terms of their influence on gastrointestinal taxa, this field relies heavily on fecalbiotics (living or once living fecal microbial populations) to quantify the effects of these compounds. In vitro fermentation models allow for quantitative analysis of specific materials and are semi-representative models of colonic fermentation [18]. Although not a complete substitute for human studies, when paired with in vivo models, in vitro analysis can be an accurate systematic approach to analyzing different parameters and end points in colonic fermentation [19].

With the recent release of the International Scientific Association for Probiotics and Prebiotics consensus statement, XOS has been categorized as a prebiotic or prebiotic candidate [3]. The prebiotic effects of XOS have previously been summarized by Broekaert et al. [20]. While there is less evidence supporting the prebiotic effects of XOS compared to other types of prebiotics, studies have shown that XOS supplementation in humans can increase SCFA and bifidobacteria, as well as improve stool consistency and frequency [21,22,23]. This paper compares the fermentation effects of XOS to previously established prebiotics (inulin and beta-glucans) in a controlled in vitro model. To the authors’ knowledge, this is the first controlled in vitro study comparing the effects of XOS to these known prebiotics. The objective of this project was to compare currently available prebiotics by their ability to change specific taxa as well as compare differences in the production of gas and common short chain fatty acids (SCFA) between these products. Inulin, XOS and beta-glucan based products were chosen for this experiment because they are established and emerging prebiotics that are commonly consumed, and offer well-demonstrated health benefits to their consumers.

## 2. Materials and Methods

### 2.1. Prebiotic Dietary Fibers Analyzed

Five common prebiotic dietary fibers were chosen for this study (Table 1), including different types of beta-glucans, inulin and xylooligosaccharide supplements.

### 2.2. Donor Information

Fecal samples were collected from three healthy volunteers (2 males, 1 female) under anaerobic conditions. Donors included individuals (ages 22–28) consuming non-specific Western diets, who do not consume any supplements, including fiber supplements. Donors were non-smokers, did not receive any antibiotic treatments in the last year, and were not affected by any known gastrointestinal diseases (Table 2).

### 2.3. Fecal Collection

Fecal samples were anaerobically collected within 5 min of the start of the fermentation (Medline Specimen Collection Kit, Medline, Inc., Rogers, MN, USA), and homogenized immediately upon collection. All data and samples collected were done in accordance with University of Minnesota policies and procedures.

### 2.4. Fermentation

The fiber samples were fermented using in vitro methods to mimic the environment of the distal colon. These methods have been used in previous in vitro studies, including Koecher et al., who found complementary results between these in vitro methods and a human intervention study of the same fibers [24]. Fiber samples (0.5 g) were hydrated in 40 mL of prepared sterile trypticase peptone fermentation media in 100 mL serum bottles, capped, and incubated for 12 h at 4 °C to limit possibility of microbial growth [25]. Following incubation, serum bottles were transferred to a circulating water bath at 37 °C for 2 h to allow the samples to reach body temperature. Post-collection, fecal samples were mixed using a 6:1 ratio of phosphate buffer solution to fecal sample. After mixing, obtained fecal slurry was combined with prepared reducing solution (2.52 g cysteine hydrochloride, 16 mL 1 N NaOH, 2.56 g sodium sulfide nonanhydride, 380 mL DD H_2_O) at a 2:15 ratio. 10 mL of the prepared fecal inoculum was added to each of the serum bottles, 0.8 mL Oxyrase^®^ was added, flushed with CO_2_, sealed, and then immediately placed in a 37 °C circulating water bath. Fecal inoculum control samples with no fiber added were prepared for SCFA and gas production comparison. Baseline pH of the fermentation media was measured, with a mean of 6.83 ± 0.04, to mimic the environment of the distal colon. Samples were prepared in triplicate and analyzed at 0, 12 and 24 h. Upon removal at each time point, total gas volume was measured. Then samples were divided into aliquots for analysis and 1 mL of copper sulfate (200 g/L) was added to cease fermentation. All samples were immediately frozen and stored at −80 °C for further analysis.

### 2.5. SCFA Analysis

SCFA samples were extracted according to Schneider et al. [26] with minor modifications, and analyzed with previously described methods [27].

### 2.6. DNA Extractions

Fecal bacteria DNA from the in vitro system were extracted using a PowerSoil DNA Isolation Kit (Mo Bio Laboratories, Inc., Carlsbad, CA, USA) following the provided operating instruction, including bead beating for 20 min.

#### 2.6.1. Primary/Secondary Amplification

The V1-V3 region of the 16S rRNA was amplified using a two-step PCR protocol. The primary amplification was done using an ABI7900 qPCR machine (Applied Biosystems, Foster City, CA, USA). The following recipe was used: 3 μL template DNA, 0.48 μL nuclease-free water, 1.2 μL 5× KAPA HiFi buffer (Kapa Biosystems, Woburn, MA, USA), 0.18 μL 10 mM dNTPs (Kapa Biosystems, Woburn, MA, USA), 0.3 μL DMSO (Fisher Scientific, Waltham, MA, USA), 0.12 μL ROX (25 μM) (Life Technologies, Carlsbad, CA, USA), 0.003 μL 1000× SYBR Green, 0.12 μL KAPA HiFi Polymerase (Kapa Biosystems, Woburn, MA, USA), 0.3 μL forward primer (10 μM), 0.3 μL reverse primer (10 μM). Cycling conditions were: 95 °C for 5 min, followed by 20 cycles of 98 °C for 20 s, 55 °C for 15 s, and 72 °C for 1 min. The primers for the primary amplification contained both 16S-specific primers (V1_27F and V3_V34R), as well as adapter tails for adding indices and Illumina flow cell adapters in a secondary amplification. The following primers were used (16S-specific sequences in bold): Meta_V1_27F (TCGTCGGCAGCGTCAGATGTGTATAAGAGACAGAGAGTTTGATCMTGGCTCAG) and Meta_V3_534R (GTCTCGTGGGCTCGGAGATGTGTATAAGAGACAGATTACCGCGGCTGCTGG).

The amplicons from the primary PCR were diluted 1:100 in sterile, nuclease-free water, and a second PCR reaction was set up to add the Illumina flow cell adapters (Illumina Inc., San Diego, CA, USA) and indices. The secondary amplification was done on a fixed block BioRad Tetrad PCR machine (Bio-Rad Laboratories, Inc., Hercules, CA, USA) using the following recipe: 5 μL template DNA, 1 μL nuclease-free water, 2 μL 5× KAPA HiFi buffer (Kapa Biosystems, Woburn, MA, USA), 0.3 μL 10 mM dNTPs (Kapa Biosystems, Woburn, MA, USA), 0.5 μL DMSO (Fisher Scientific, Waltham, MA, USA) 0.2 μL KAPA HiFi Polymerase (Kapa Biosystems, Woburn, MA, USA), 0.5 μL forward primer (10 μM), 0.5 μL reverse primer (10 μM). Cycling conditions were: 95 °C for 5 min, followed by 10 cycles of 98 °C for 20 s, 55 °C for 15 s, 72 °C for 1 min, followed by a final extension at 72 °C for 10 min. The following indexing primers were used (X indicates the positions of the 8 bp indices): Forward indexing primer: AATGATACGGCGACCACCGAGATCTACACXXXXXXXXTCGTCGGCAGCGTC and Reverse indexing primer: CAAGCAGAAGACGGCATACGAGATXXXXXXXXGTCTCGTGGGCTCGG.

#### 2.6.2. Normalization and Sequencing

The samples were normalized using a SequalPrep capture-resin bead plate (Life Technologies, Carlsbad, CA, USA) and pooled using equal volume. The final pools were quantified via PicoGreen dsDNA assay (Life Technologies, Carlsbad, CA, USA) and diluted to 2 nM. 10 μL of the 2 nM pool was denatured with 10 μL of 0.2 N NaOH, diluted to 8 pM in Illumina’s HT1 buffer, spiked with 15% phiX, heat denatured at 96 °C for 2 min, and sequenced using a MiSeq 600 cycle v3 kit (Illumina, San Diego, CA, USA).

#### 2.6.3. Sequence Processing and Analysis

Generated sequence data was processed and analyzed using QIIME [28]. Fastq sequence data was processed with the University of Minnesota’s gopher-pipeline for metagenomics [29]. Sequence data had adapters removed and sliding quality trimming window by Trimmomatic [30]; primers removed and overlapping reads merged by Pandaseq [31]. Within QIIME, chimera checking done by chimera slayer, Open reference OTU picking completed with Usearch61, taxonomic identification using GreenGenes (Version 13.8) reference database, rarefied to 14,393 sequences per sample. Analysis was performed using *R* (*R* Development Core Team, Vienna, Austria, 2012).

### 2.7. Statistical Analysis

All statistical analysis was performed using *R* software (Version 3.2.2, *R* Development Core Team, Vienna, Austria, 2012). Differences in means were determined using the Kruskal-Wallis ANOVA test, testing the null hypothesis that the location parameter of the groups of abundancies for a given OTU is the same. Multiple comparisons were corrected using the Benjamini-Hochberg FDR (false discovery rate) procedure for multiple comparisons. For gas and SCFA data, ANOVA with Tukey HSD was used to compare means. Significance was set for *p*-values < 0.05 for all statistical tests.

### 2.8. Consent Ethics Approval Code

Voluntary informed consent was obtained from all fecal donors prior to this study according to University of Minnesota policies and procedures.

## 3. Results

### 3.1. Gas Production

**Figure 1:**
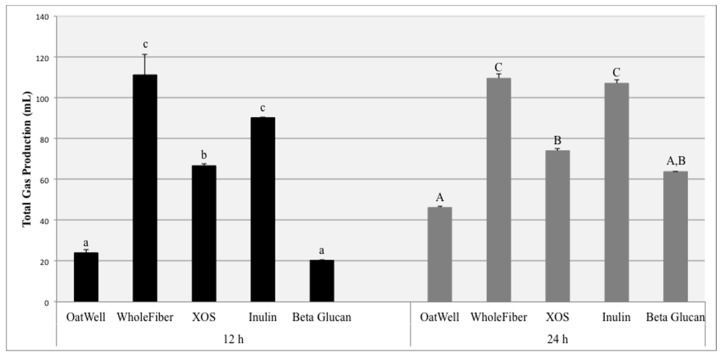
Total gas production comparing fermentation differences among five prebiotic dietary fibers for three individuals at 12 h and 24 h post-exposure to fecal microbiota in an in vitro fermentation system. Data displayed are means (3 donors × 3 replicates = 9) for each prebiotic dietary fiber ± SD. Columns with different letters are significantly different from one another within each time measurement (lowercase: 12 h; uppercase: 24 h). Data were analyzed using ANOVA with Tukey HSD (*p* < 0.05).

At 12 h, the OatWell and the pure beta-glucan samples produced similar amounts of total gas (Figure 1). The XOS samples produced significantly more gas than the pure beta glucan samples (*p* < 0.01) or the OatWell samples (*p* < 0.01). The WholeFiber and pure inulin samples produced similar amounts of total gas (*p* = 0.102), and the total gas production for both of these prebiotic dietary fibers was significantly higher than the XOS samples, (*p* < 0.01 and *p* = 0.045), respectively. At 24 h, the OatWell samples had the lowest gas production (46.2 mL) and were similar to the pure beta-glucan samples (63.7 mL; *p* = 0.498). The 24 h XOS samples (74.0 mL) were also similar to the beta-glucan samples (*p* = 0.926). However, the 24 h WholeFiber (109.6 mL) and pure inulin (107.1 mL) samples produced significantly more gas than XOS, beta-glucan and Oatwell samples (*p* < 0.01). Individual variation in gas production can be seen in Table S1.

### 3.2. SCFA Production

For all SCFA analysis, analysis at 12 and 24 h shows production only, from baseline corrected samples. Acetate, propionate and butyrate production is shown as μmol/mL of fermentation media. Individual variation in SCFA production can be seen in Table S1.

**Figure 2:**
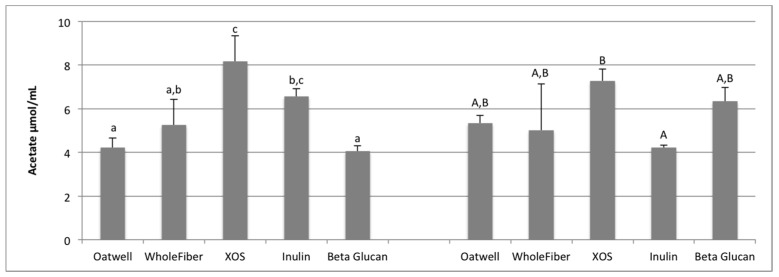
Acetate production at 12 h and 24 h of fermentation for five prebiotic dietary fibers displayed as μmol/mL of fermentation inoculum. Data displayed are means (3 donors × 3 replicates = 9) for each prebiotic dietary fiber ± SD. Columns with different letters are significantly different from one another (lowercase: 12 h; uppercase: 24 h). Data were analyzed using ANOVA with Tukey HSD (*p* < 0.05).

Acetate production at 12 h was similar for the Oatwell, WholeFiber and beta-glucan samples (Figure 2). The XOS samples produced significantly more acetate at 12 h than the Oatwell, WholeFiber or beta-glucan samples (*p* < 0.05). The inulin samples had similar amounts of acetate compared to the WholeFiber and XOS samples, and significantly more than the Oatwell (*p* = 0.024) and beta-glucan (*p* = 0.013) samples at 12 h. After 24 h, the inulin samples contained less acetate than the XOS samples (*p* = 0.038), while the Oatwell, WholeFiber and beta-glucan samples were similar to both the XOS and inulin samples.

**Figure 3:**
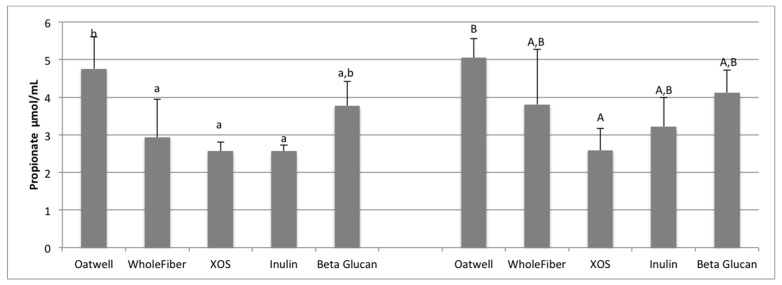
Propionate production at 12 h and 24 h of fermentation for five prebiotic dietary fibers displayed as μmol/mL of fermentation inoculum. Data displayed are means (3 donors × 3 replicates = 9) for each prebiotic dietary fiber ± SD. Columns with different letters are significantly different from one another (lowercase: 12 h; uppercase: 24 h). Data were analyzed using ANOVA with Tukey HSD (*p* < 0.05).

Propionate production at 12 h of fermentation was highest for the OatWell samples (4.76 μmol/mL) and was significantly greater than the WholeFiber (*p* = 0.029), XOS (*p* = 0.005) and inulin samples (*p* = 0.004), and similar to the beta-glucan samples (Figure 3). At 24 h of fermentation, the Oatwell samples had the highest mean production 5.05 μmol/mL, which was significantly greater than the XOS samples (2.58 μmol/mL; *p* = 0.021), and similar to WholeFiber, inulin and beta-glucan samples.

**Figure 4:**
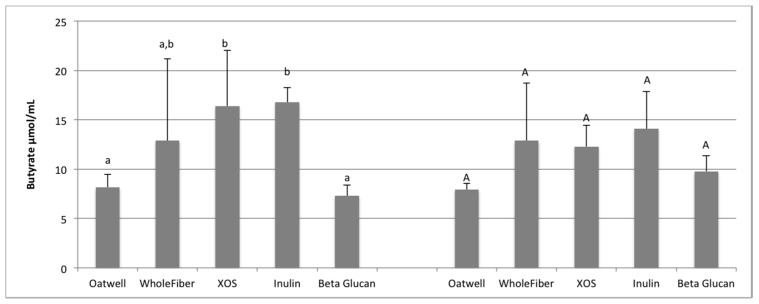
Butyrate production at 12 h and 24 h of fermentation for five prebiotic dietary fibers displayed as μmol/mL of fermentation inoculum. Data displayed are means (3 donors × 3 replicates = 9) for each prebiotic dietary fiber ± SD. Columns with different letters are significantly different from one another (lowercase: 24 h; uppercase: 24 h). Data were analyzed using ANOVA with Tukey HSD (*p* < 0.05).

Butyrate production after 12 h of fermentation ranged from 7.30 μmol/mL for the beta-glucan samples to 16.76 μmol/mL for the inulin samples (Figure 4). The inulin samples had the highest average production, and were similar to the XOS (16.38 μmol/mL) and WholeFiber samples (12.89 μmol/mL). The XOS samples were significantly higher than the Oatwell (*p* = 0.035) and beta-glucan samples (*p* = 0.014). At 24 h of fermentation, all five prebiotic dietary fibers were statistically similar to one another, ranging from 7.93–14.08 μmol/mL due to a wide ranges in response differences between the three fecal donors used in this study.

### 3.3. Microbiota Analysis

Extracted DNA from in vitro samples were sequenced using the MiSeq Illumina platforms (Illumina Inc., San Diego, CA, USA) generating a total of 31,591,899 sequence reads. Sequencing parameters identified reads belonging to 11 bacterial phyla, 61 families and 97 genera.

For all three donors, the phyla Bacteroidetes and Firmicutes represented > 80% of all sequence reads (Figure S1) across 24 h of fermentation. At the family level, 13 families represented 85% of all sequence reads (Figure S2), while 11 genera represented >75% of all sequence reads (Figure S3). Six metrics measuring α-diversity for all donors showed various degrees of similarity by donors (Figure S4), and by treatment (Figure S5). Both Unifrac and Bray-Curtis β-diversity metrics (measuring pairwise dissimilarity between samples), showed similarity among technical replicates of treatment groups for each donor (Figure S6) as well as for all treatment groups for each respective donor (Figure S7).

After 24 h of fermentation, the Oatwell samples significantly decreased the population of SMB53 (0 h:9.11 OTUs; 24 h:2.11 OTUs; *p* = 0.008), Lachnospira and Faecalibacterium (0 h:26.56 OTUs; 24 h:4.44 OTUs; *p* = 0.008 and 0 h 136.44 OTUs; 24 h:66 OTUs; *p* = 0.022, respectively) (Table 3). No genera analyzed showed significant increases in 24 h for the Oatwell samples measured for the three fecal donors in this study. The WholeFiber samples (Table 4) significantly increased the genus Collinsella at 24 h compared to 0 h (0 h:68 OTUs; 24 h:299.78 OTUs; *p* = 0.011). Bifidobacterium populations were only significantly increased at 24 h compared to 0 h for the XOS samples (0 h:0.67 OTUs; 24 h:5.22 OTUs; *p* = 0.038), while the same samples showed a significant decrease in Lachnospira and Faecalibacterium (*p* = 0.038 and *p* = 0.03) (Table 5). The inulin samples (Table 6) increased Collinsella (0 h:55.11 OTUs; 24 h:291.44 OTUs; *p* = 0.016). The pure beta glucan samples significantly decreased Lachnospira and Faecalibacterium (*p* = 0.008) (Table 7).

## 4. Discussion

The aim of this study was to investigate the beneficial effects of commonly consumed prebiotic dietary fibers, including their ability to influence the growth of identified bacterial populations, form beneficial SCFAs, and the amount of gas they produce due to fermentation. Total gas production due to fiber fermentation depends on a wide range of factors. The inulin samples and the WholeFiber samples (mixture of dried chicory root inulin, pectin and hemi/cellulose) resulted in the highest gas production at both 12 and 24 h. These results are consistent with results from both clinical feeding studies and other in vitro experiments, in which fermentation of inulin products resulted in high amounts of gas production, sometimes resulting in mild negative gastrointestinal symptoms, depending on the dosage [32,33]. Similar in vitro studies have found inulin to be much more fermentable than beta-glucan products, for both barley and oat-derived beta-glucans [34]. XOS fermentation results in less gas production than the inulin products, and more gas than beta-glucan products. Because of these findings, previous studies based on digestive tolerance and parameters have established a tolerated daily dosage for XOS of approximately 12 g/day [35].

SCFA production due to the fermentation of prebiotic dietary fibers promotes many beneficial health outcomes to the host. SCFA production may contribute to up to 10% of the host’s metabolizable energy daily, with production of total SCFAs usually between 100–200 mmol/day, but is highly dependent on the donor and availability of substrates for fermentation [36,37]. At 12 h of fermentation, the OatWell and beta-glucan samples had significantly higher concentrations of propionate, and the highest mean concentration at 24 h, compared to the other prebiotic dietary fibers analyzed. Similar in vitro studies with beta-glucan based products have also shown similar preference for these products to result in propionate formation [34]. Although no mechanism has been identified, and studies show conflicting results [38], elevated serum propionate concentrations have been shown to have a hypocholesterolaemic effect [39]. Propionate may also play an influential role in satiety, although mechanisms still remain unclear [40,41]. Cholesterol-lowering properties of beta-glucans may be limited to effects from the upper-GI, although many propionate-producing bacteria have a preference to fermenting various types of beta-glucans (*Bacteroides*, *Prevotella*, *Clostridium*) based on the presence of genes responsible for endo-β-glucanase enzyme production [42].

Microbial diversity among fecal donors complicates the identification of trends among the five treatment groups (Supplemental Figures S8 and S9). In terms of taxonomic shift, the inulin-based products were fermented nearly identically by all three fecal donors. Both pure inulin and WholeFiber promoted the growth of *Collinsella* comparing the 24 h samples to the 0 h samples. Inulin-type fructans have been shown in clinical studies to promote substantial growth of *Collinsella*, paralleled with increased urinary hippurate levels [43]. Hippurate is a metabolite derived from various fermentation processes in the gut that has been found in decreased concentrations in obese individuals compared to lean individuals, and also between diabetics and non-diabetics [44,45,46]. The genus *Collinsella* has been found in lower concentrations in individuals with IBD compared to healthy controls [47], while *Collinsella aerofaciens* has been associated with low risk of colorectal cancer [48]. Increases in *Collinsella* and increased urinary hippurate levels are considered a beneficial effect of inulin consumption due to its prebiotic capacity [43]. In vivo studies with inulin, scFOS and resistant starch supplementation have found decreases in the SMB53 genus, consistent with the OatWell treatment in the present study [30,31].

A significant increase in the genus *Bifidobacterium* was observed only with the XOS treatment. Rycroft et al. found a similar affinity of *Bifidobacterium* toward XOS [49]. However, previous studies have shown inulin to also stimulate the growth of *Bifidobacterium* [49,50,51,52]. While there was an overall rise in *Bifidobacterium* with the inulin treatment group between 0 h and 24 h (1.33, 5.44 OTUs respectively), this study did not find that increase to be significant (*p* = 0.304). The small sample size and individual microbiome variability likely played a role in this result. Increases in *Bifidobacterium* have been heavily studied and reviewed, and are considered a beneficial effect due to their correlation with many positive health outcomes [1]. *Bifidobacteria* reside naturally in the gastrointestinal tract of healthy human adults and have a strong affinity to ferment oligosaccharides, making them a common marker for prebiotic capacity. *Bifidobacterium* is a unique genus of bacteria in that no gas is formed as an end product of metabolism [53]. Like *Lactobacillus*, these bacteria are saccharolytic, often considered a beneficial trait [54]. *Bifidobacteria* also do not produce any known carcinogenic substances in vivo. *Bifidobacteria* concentrations have been negatively associated with obesity and weight gain [55,56,57,58]. Increases in *Bifidobacteria* have also been correlated with a decrease in blood lipopolysaccharides (LPS), inflammatory reagents that play a role in the development of inflammatory metabolic disorders and conditions, and are primarily found in gram-negative bacteria [59]. LPS induce the activation of Toll-like receptor 4 (TLR4), which leads to inflammation due to release of pro-inflammatory cytokines and chemokines [60].

In vitro fermentations are semi-representative models of colonic fermentation, but have limitations [18]. This study did not include an in vitro digestion process, which would remove digestible contents from the samples prior to fermentation, and would be a more representative model. However, because the test substrates are primarily fiber, which is non-digestible, this should have minimal impact on the results of this study. In vivo, formed gases are continually absorbed and colonic absorption is rapid. Because SCFAs are rapidly absorbed and difficult to measure, in vitro models help to understand the kinetics of colonic fermentation. However, in vitro models must be paired with similar in vivo models to better understand the full mechanisms of action resulting from colonic fermentation of prebiotic dietary fibers. Because the SCFA are not absorbed in in vitro models, the SCFA produced can alter the pH of the fermentation media. While the fermentation media was designed to mimic the pH of the distal colon at baseline, the pH of the media in this study was not further controlled throughout the experiment. This is another limitation of this in vitro model. An additional limitation of this study was the small sample size. The present study was conducted using fecal inoculum from only three donors. Due to the individual variability between the donor’s microbiota (Figure S7), a larger sample size may be needed to achieve a more representative view of the effects of each of the fibers.

## 5. Conclusions

All five prebiotics measured in this study display fermentability and SCFA production that could have potential health benefits. Depending on their structure, each compound offers a specific carbon source for fermentation by different bacterial populations, yielding changes in beneficial taxa and production of various amounts of SCFAs and gas in vitro. For instance, while OatWell and beta-glucans promoted propionate production, XOS increased concentrations of *Bifodobacterium*, and WholeFiber and pure inulin promoted *Collinsella* growth. Findings in this study are consistent with other in vitro studies with similar prebiotic dietary fibers, as well as clinical feeding studies [34,43,49,61,62,63].

## Figures and Tables

**Table 1:** Comparison Prebiotic Dietary Fibers Analyzed with in vitro Fermentation System.

Prebiotic Dietary Fibers	Supplier Information
OatWell (Oatbran containing 28% beta-glucan)	DSM Nutritional Products, Ltd. (Kaiseraugust, Switzerland)
WholeFiber (A dried chicory root blend containing: inulin, pectin, hemi/cellulose)	WholeFiber, Inc. (Pennington, NJ, USA)
Xylooligosaccharide (XOS)	AIDP, Inc. (Industry, CA, USA)
Pure Inulin	Cargill, Inc. (Wayzata, MN, USA)
Pure Beta-glucan	Megazyme, Inc. (Bray, Wicklow, Ireland)

**Table 2:** Demographic Characteristics of Three Fecal Donors.

Demographic characteristics	Donor 1	Donor 2	Donor 3
Age	26	25	22
Sex	Female	Male	Male
Body Mass Index (kg/m^2^)	28.1	26.3	23.0

**Table 3:** Combined changes across 24 h of fermentation for Oatwell samples of identified genera ^1^.

*Genera*	0 h	24 h	*p*-Value
Actinobacteria			
*Bifidobacterium*	1.22	0.89	0.660
*Adlercreutzia*	1.44	3.00	0.470
*Collinsella*	48.44	140.56	0.089
Bacteroidetes			
*Alistipes*	2.56	1.33	0.674
*Parabacteroides*	135.00	155.89	0.952
*Bacteroides*	755.67	865.00	0.192
Firmicutes			
*Eubacterium*	0.44	0.56	0.817
*Veillonella*	1.11	1.22	0.980
*Dorea*	2.33	3.56	0.516
*Acidaminococcus*	3.22	10.44	0.817
*Clostridium*	7.67	8.33	0.769
*Anaerostipes*	8.11	6.00	0.674
*Turicibacter*	8.67	1.22	0.286
*SMB53*	9.11	2.11	0.008 *
*Ruminococcus*	11.22	23.22	0.263
*Lactococcus*	11.67	10.67	0.980
*Streptococcus*	15.22	8.11	0.511
*Roseburia*	20.22	22.33	0.980
*Oscillospira*	21.78	36.67	0.121
*Lachnospira*	26.56	4.44	0.008 *
*Phascolarctobacterium*	27.78	173.33	0.263
*Dialister*	39.56	43.00	0.560
*Blautia*	41.89	53.11	0.470
*Coprococcus*	49.89	39.00	0.396
*Ruminococcus*	61.33	40.67	0.289
*Faecalibacterium*	136.44	66.00	0.022 *
Proteobacteria			
*Escherichia*	0.44	1.44	0.325
*Haemophilus*	10.22	0.67	0.286
*Sutterella*	10.78	14.44	0.980
*Bilophila*	13.67	14.78	0.788
Verrucomicrobia			
*Akkermansia*	5.00	12.00	0.980

^1^ Replicate samples were pooled among donor at each respective time point (3 donors × 3 replicated = 9). Samples were analyzed between differentially represented OTUs for significant changes after 24 h of fermentation compared to 0 h samples. Values are the number of OTUs after rarefaction to 3668 sequences per sample. Data were analyzed using the Kruskal-Wallis ANOVA test, with the FDR (false discovery rate) multiple comparisons correction. * Indicates significance at *p* ≤ 0.05.

**Table 4:** Combined changes across 24 h of fermentation for WholeFiber samples of identified genera ^1^.

*Genera*	0 h	24 h	*p*-Value
Actinobacteria			
*Adlercreutzia*	0.89	3.89	0.239
*Bifidobacterium*	1.11	1.11	0.785
*Collinsella*	68.00	299.78	0.011 *
Bacteroidetes			
*Alistipes*	1.11	0.56	0.894
*Parabacteroides*	131.44	142.00	0.913
*Bacteroides*	743.56	776.56	0.785
Firmicutes			
*Eubacterium*	1.11	0.78	0.799
*Veillonella*	1.22	1.00	0.960
*Dorea*	2.00	5.00	0.785
*Acidaminococcus*	2.67	11.33	0.894
*SMB53*	5.67	4.00	0.239
*Clostridium*	7.33	13.22	0.896
*Anaerostipes*	10.22	1.22	0.239
*Ruminococcus*	10.89	19.67	0.943
*Streptococcus*	12.67	8.78	0.785
*Turicibacter*	14.22	2.44	0.960
*Lachnospira*	14.78	72.00	0.237
*Oscillospira*	17.22	14.89	0.647
*Lactococcus*	20.44	9.22	0.896
*Phascolarctobacterium*	24.67	60.44	0.501
*Dialister*	26.11	58.22	0.943
*Roseburia*	28.56	6.00	0.674
*Blautia*	32.44	49.44	0.156
*Coprococcus*	45.78	66.44	0.501
*Ruminococcus*	54.22	39.33	0.261
*Faecalibacterium*	154.89	93.11	0.080
Proteobacteria			
*Escherichia*	0.78	1.44	0.960
*Sutterella*	4.00	32.44	0.894
*Haemophilus*	10.67	0.56	0.107
*Bilophila*	10.67	7.67	0.896
Verrucomicrobia			
*Akkermansia*	17.00	3.33	0.501

^1^ Replicate samples were pooled among donor at each respective time point (3 donors × 3 replicated = 9). Samples were analyzed between differentially represented OTUs for significant changes after 24 h of fermentation compared to 0 h samples. Values are the number of OTUs after rarefaction to 3668 sequences per sample. Data were analyzed using the Kruskal-Wallis ANOVA test, with the FDR multiple comparisons correction. * Indicates significance at *p* ≤ 0.05.

**Table 5:** Combined changes across 24 h of fermentation for xylooligosaccharide samples of identified genera ^1^.

*Genera*	0 h	24 h	*p*-Value
Actinobacteria			
*Bifidobacterium*	0.67	5.22	0.038 *
*Adlercreutzia*	1.33	1.78	0.972
*Collinsella*	58.44	154.00	0.413
Bacteroidetes			
*Alistipes*	1.44	0.56	0.413
*Parabacteroides*	147.33	133.33	0.972
*Bacteroides*	770.89	870.44	0.189
Firmicutes			
*Eubacterium*	0.33	1.67	0.364
*Veillonella*	0.67	0.00	0.162
*Acidaminococcus*	1.33	2.33	0.972
*Dorea*	2.11	3.67	0.423
*SMB53*	7.33	5.44	0.558
*Anaerostipes*	7.44	3.44	0.447
*Turicibacter*	8.00	8.56	0.972
*Clostridium*	8.44	4.00	0.087
*Ruminococcus*	12.78	26.11	0.087
*Streptococcus*	14.11	4.67	0.367
*Lachnospira*	21.11	5.33	0.038 *
*Oscillospira*	21.33	21.78	0.972
*Phascolarctobacterium*	23.44	16.33	0.972
*Lactococcus*	23.89	21.00	0.982
*Roseburia*	28.89	35.33	0.972
*Dialister*	33.89	41.56	0.831
*Blautia*	39.22	65.00	0.087
*Ruminococcus*	45.11	37.33	0.385
*Coprococcus*	47.11	48.67	0.705
*Faecalibacterium*	148.56	79.56	0.030 *
Proteobacteria			
*Escherichia*	0.89	0.44	0.972
*Haemophilus*	6.44	3.11	0.972
*Bilophila*	17.78	6.22	0.107
*Sutterella*	25.78	40.89	0.831
Verrucomicrobia			
*Akkermansia*	2.78	5.00	0.841

^1^ Replicate samples were pooled among donor at each respective time point (3 donors × 3 replicated = 9). Samples were analyzed between differentially represented OTUs for significant changes after 24 h of fermentation compared to 0 h samples. Values are the number of OTUs after rarefaction to 3668 sequences per sample. Data were analyzed using the Kruskal-Wallis ANOVA test, with the FDR multiple comparisons correction. * Indicates significance at *p* ≤ 0.05.

**Table 6:** Combined changes across 24 h of fermentation for pure inulin samples of identified genera ^1^.

*Genera*	0 h	24 h	*p*-Value
Actinobacteria			
*Bifidobacterium*	1.33	5.44	0.304
*Adlercreutzia*	1.33	2.00	0.845
*Collinsella*	55.11	291.44	0.016 *
Bacteroidetes			
*Alistipes*	1.56	0.89	0.878
*Parabacteroides*	147.44	164.78	0.887
*Bacteroides*	726.78	644.44	0.652
Firmicutes			
*Veillonella*	0.78	0.56	0.908
*Eubacterium*	0.89	1.56	0.908
*Dorea*	1.78	7.00	0.640
*Acidaminococcus*	3.11	18.67	0.887
*SMB53*	7.44	9.11	0.965
*Turicibacter*	7.78	4.89	0.652
*Clostridium*	8.22	7.11	0.845
*Ruminococcus*	9.56	34.11	0.309
*Anaerostipes*	11.22	4.67	0.652
*Streptococcus*	13.00	12.44	0.887
*Lactococcus*	19.11	9.67	0.908
*Lachnospira*	21.00	4.89	0.022 *
*Phascolarctobacterium*	26.22	21.00	0.887
*Oscillospira*	26.33	10.11	0.034 *
*Roseburia*	26.78	14.11	0.887
*Dialister*	32.67	95.11	0.887
*Blautia*	38.22	50.22	0.690
*Coprococcus*	48.11	60.89	0.640
*Ruminococcus*	52.33	43.00	0.908
*Faecalibacterium*	148.11	187.33	0.652
Proteobacteria			
*Escherichia*	1.00	1.22	0.908
*Haemophilus*	9.11	2.67	0.652
*Sutterella*	14.00	31.22	0.908
*Bilophila*	16.89	7.78	0.309
Verrucomicrobia			
*Akkermansia*	7.78	7.44	0.304

^1^ Replicate samples were pooled among donor at each respective time point (3 donors × 3 replicated = 9). Samples were analyzed between differentially represented OTUs for significant changes after 24 h of fermentation compared to 0 h samples. Values are the number of OTUs after rarefaction to 3668 sequences per sample. Data were analyzed using the Kruskal-Wallis ANOVA test, with the FDR multiple comparisons correction. * Indicates significance at *p* ≤ 0.05.

**Table 7:** Combined changes across 24 h of fermentation for pure beta-glucan samples of identified genera ^1^.

*Genera*	0 h	24 h	*p*-Value
Actinobacteria			
*Bifidobacterium*	0.33	0.33	1.000
*Adlercreutzia*	2.00	1.89	0.843
*Collinsella*	69.22	85.11	0.723
Bacteroidetes			
*Alistipes*	0.78	0.89	0.778
*Parabacteroides*	119.56	179.78	0.778
*Bacteroides*	776.11	854.33	0.664
Firmicutes			
*Eubacterium*	0.11	0.44	0.778
*Veillonella*	0.56	0.22	0.778
*Dorea*	0.89	3.11	0.110
*Acidaminococcus*	2.33	15.11	0.778
*SMB53*	6.11	4.89	0.778
*Lactococcus*	6.11	0.67	0.778
*Anaerostipes*	7.44	5.22	0.778
*Turicibacter*	8.11	3.00	0.803
*Ruminococcus*	9.44	18.67	0.166
*Clostridium*	10.11	3.33	0.110
*Streptococcus*	14.89	6.44	0.256
*Roseburia*	16.11	54.33	0.510
*Lachnospira*	21.22	3.89	0.008 *
*Oscillospira*	24.33	35.11	0.389
*Phascolarctobacterium*	29.00	125.33	0.283
*Dialister*	30.56	43.67	0.819
*Coprococcus*	44.11	20.78	0.211
*Blautia*	45.11	68.11	0.408
*Ruminococcus*	59.67	44.44	0.500
*Faecalibacterium*	152.11	62.67	0.008 *
Proteobacteria			
*Escherichia*	0.89	0.56	0.778
*Haemophilus*	11.00	0.78	0.110
*Sutterella*	14.00	35.44	0.778
*Bilophila*	14.44	13.89	0.778
Verrucomicrobia			
*Akkermansia*	9.11	15.89	0.778

^1^ Replicate samples were pooled among donor at each respective time point (3 donors × 3 replicated = 9). Samples were analyzed between differentially represented OTUs for significant changes after 24 h of fermentation compared to 0 h samples. Values are the number of OTUs after rarefaction to 3668 sequences per sample. Data were analyzed using the Kruskal-Wallis ANOVA test, with the FDR multiple comparisons correction. * Indicates significance at *p* ≤ 0.05.

## Supplementary Materials

The following are available online at www.mdpi.com/2072-6643/9/12/1361/s1. Table S1: Gas and SCFA Production Comparing Fermentation Differences of Five Prebiotic Dietary Fibers at 12 h and 24 h Post-Exposure to Fecal Microbiota from Three Individual Donors in an In Vitro Fermentation System, Figure S1: Identified phyla from three fecal donors microbiota at 0, 12 and 24 h of fermentation for five prebiotic dietary fibers analyzed based on percent of sequence reads, Figure S2: Identified abundant families for three fecal donors at 0, 12 and 24 h of fermentation for five prebiotic dietary fibers analyzed based on percent of sequence reads, Figure S3: Identified abundant genera for three fecal donors at 0, 12 and 24 h of fermentation for five prebiotic dietary fibers analyzed based on percent of sequence reads, Figure S4: Six metrics of analysis for alpha-diversity among samples at 0, 12 and 24 h of analysis, grouped by donor for all five prebiotic dietary fibers analyzed, Figure S5: Six metrics of analysis for alpha-diversity among samples at 0, 12 and 24 h of analysis, grouped by treatment for all three fecal donors, Figure S6: Bray-Curtis β-diversity principal component analysis of technical replicates among each treatment group between microbiota analysis of three fecal donors, Figure S7: Bray-Curtis β-diversity principal component analysis among microbiota of three fecal donors at 0, 12 and 24 h of analysis, Figure S8: Variations in abundant phyla among three donors analyzed, Figure S9: Variations in treatment groups and pooled donors analyzed.
