# Supplementary material for: Prebiotic Dietary Fiber and Gut Health: Comparing the in Vitro Fermentations of Beta-Glucan, Inulin and Xylooligosaccharide
Source: Nutrients. 2017 Dec 15;9(12):1361. doi: 10.3390/nu9121361 (PMC5748811; doi:10.3390/nu9121361)

**Supplementary Materials:**

Table S1. Gas and SCFA Production Comparing Fermentation Differences of Five Prebiotic Dietary Fibers at 12 h and 24 h Post-Exposure to Fecal Microbiota from Three Individual Donors in an *In Vitro* Fermentation System.

|  | Donor 1 | | | | Donor 2 | | | | Donor 3 | | | |
| --- | --- | --- | --- | --- | --- | --- | --- | --- | --- | --- | --- | --- |
|  | 12 h | | 24h | | 12h | | 24h | | 12h | | 24h | |
|  | Mean | SD | Mean | SD | Mean | SD | Mean | SD | Mean | SD | Mean | SD |
|  | **Gas (mL)** | | | | | | | | | | | |
| Oatwell | 33.33 | 4.04 | 54.33 | 2.08 | 21.33 | 3.05 | 39.0 | 1.0 | 17.33 | 1.15 | 45.33 | 2.31 |
| WholeFiber | 113.33 | 5.77 | 133.33 | 5.77 | 102.0 | 7.0 | 133.33 | 5.77 | 76.33 | 23.62 | 62.0 | 2.0 |
| XOS | 72.33 | 3.21 | 68.0 | 4.0 | 76.33 | 2.31 | 84.0 | 2.0 | 31.33 | 1.52 | 70.0 | 4.0 |
| Inulin | 110.67 | 1.15 | 136.67 | 2.88 | 82.33 | 1.52 | 100.67 | 2.31 | 77.67 | 1.53 | 84.0 | 5.29 |
| Beta Glucan | 36.0 | 1.0 | 61.33 | 1.15 | 18.0 | 1.73 | 70.67 | 1.15 | 7.0 | 1.73 | 59.33 | 1.15 |
|  | **Acetate (μmol/mL)** | | | | | | | | | | | |
| Oatwell | 4.45 | 0.25 | 4.66 | 0.37 | 4.34 | 0.83 | 5.74 | 4.38 | 3.85 | 0.28 | 5.64 | 0.33 |
| WholeFiber | 4.74 | 1.37 | 2.17 | 0.61 | 5.52 | 1.6 | 6.9 | 5.67 | 5.53 | 0.51 | 5.99 | 0.03 |
| XOS | 9.41 | 1.45 | 5.41 | 0.09 | 8.59 | 1.61 | 7.82 | 0.13 | 6.81 | 0.44 | 8.62 | 1.39 |
| Inulin | 5.11 | 0.22 | 3.79 | 0.21 | 4.62 | 0.36 | 3.34 | 0.12 | 9.98 | 0.47 | 5.53 | 0.04 |
| Beta Glucan | 4.63 | 0.31 | 6.69 | 0.5 | 4.29 | 0.17 | 6.28 | 0.79 | 3.25 | 0.27 | 6.08 | 0.62 |
|  | **Propionate (μmol/mL)** | | | | | | | | | | | |
| Oatwell | 3.51 | 0.83 | 3.84 | 0.03 | 4.82 | 0.52 | 4.79 | 1.4 | 5.95 | 1.18 | 6.33 | 0.1 |
| WholeFiber | 2.18 | 0.93 | 2.09 | 1.08 | 4.21 | 1.33 | 4.24 | 1.65 | 2.11 | 0.81 | 5.1 | 1.64 |
| XOS | 1.91 | 0.29 | 1.09 | 0.97 | 4.08 | 0.11 | 3.27 | 0.71 | 1.72 | 0.32 | 2.59 | 0.08 |
| Inulin | 1.35 | 0.09 | 4.23 | 0.83 | 3.32 | 0.22 | 3.9 | 1.31 | 3.02 | 0.2 | 1.54 | 0.15 |
| Beta Glucan | 2.45 | 0.21 | 3.54 | 0.02 | 4.36 | 0.62 | 4.14 | 0.46 | 4.52 | 1.12 | 4.69 | 1.29 |
|  | **Butyrate (μmol/mL)** | | | | | | | | | | | |
| Oatwell | 11.68 | 0.76 | 9.75 | 0.33 | 7.81 | 2.8 | 5.8 | 0.73 | 5.01 | 0.33 | 8.25 | 0.59 |
| WholeFiber | 12.59 | 8.78 | 12.11 | 3.01 | 13.18 | 9.31 | 19.89 | 12.91 | 12.91 | 6.71 | 6.67 | 1.15 |
| XOS | 20.6 | 6.57 | 10.14 | 2.23 | 14.72 | 7.76 | 16.17 | 0.3 | 13.84 | 2.75 | 10.55 | 3.6 |
| Inulin | 20.48 | 1.33 | 13.58 | 8.22 | 20.09 | 2.37 | 17.0 | 1.26 | 9.73 | 0.81 | 11.67 | 1.89 |
| Beta Glucan | 10.46 | 1.38 | 12.64 | 0.5 | 7.7 | 0.81 | 8.53 | 2.21 | 3.75 | 1.00 | 8.17 | 2.08 |

Data displayed are means and SD (3 replicates per donor) for each prebiotic dietary fiber

Figure S1. Identified phyla from three fecal donors microbiota at 0, 12 and 24 h of fermentation for five prebiotic dietary fibers analyzed based on percent of sequence reads.


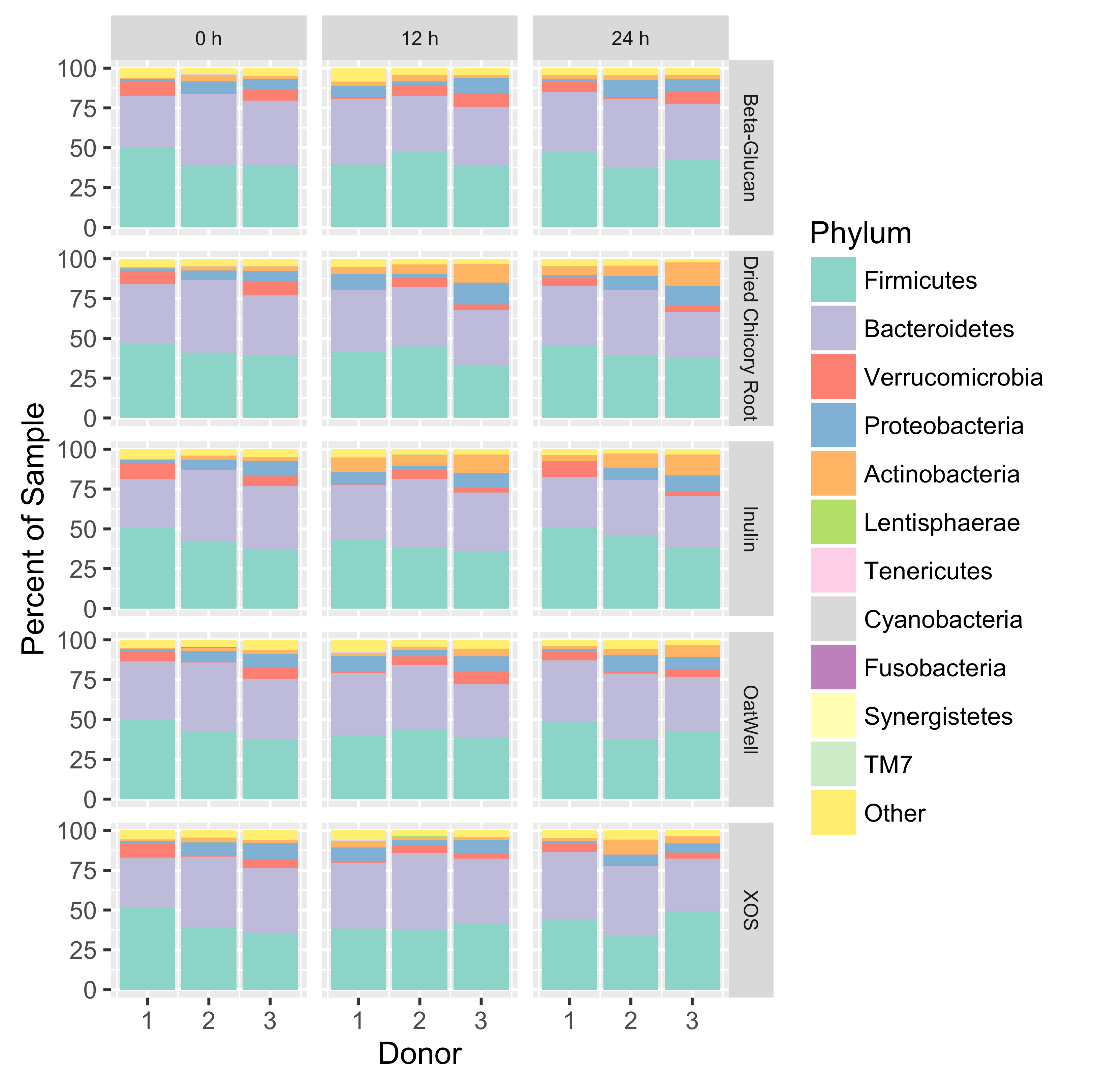


Figure S2. Identified abundant families for three fecal donors at 0, 12 and 24 h of fermentation for five prebiotic dietary fibers analyzed based on percent of sequence reads.


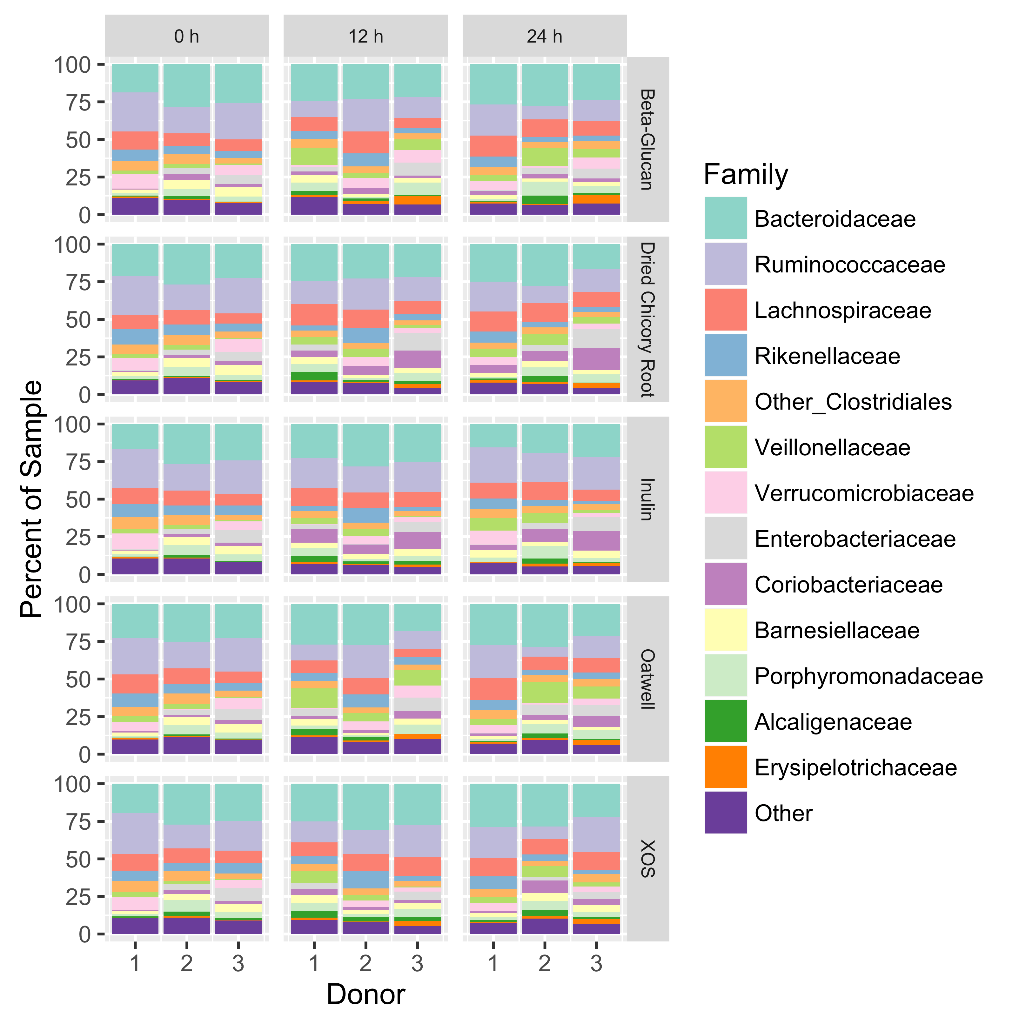


Other designates <1% average per sample.

Figure S3. Identified abundant genera for three fecal donors at 0, 12 and 24 h of fermentation for five prebiotic dietary fibers analyzed based on percent of sequence reads.


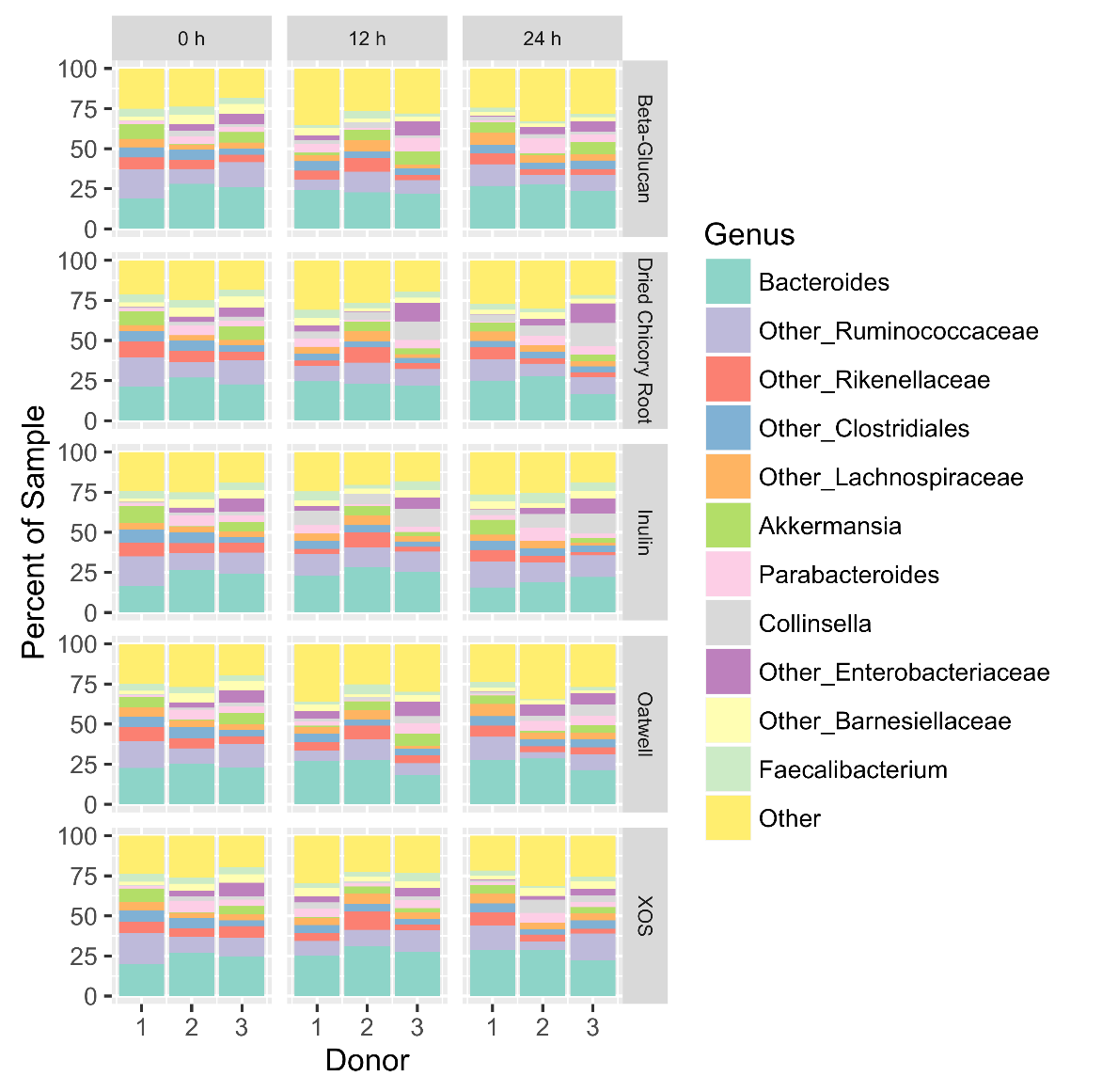


Other designates <2% average per sample.

Figure S4. Six metrics of analysis for alpha-diversity among samples at 0, 12 and 24 h of analysis, grouped by donor for all five prebiotic dietary fibers analyzed.


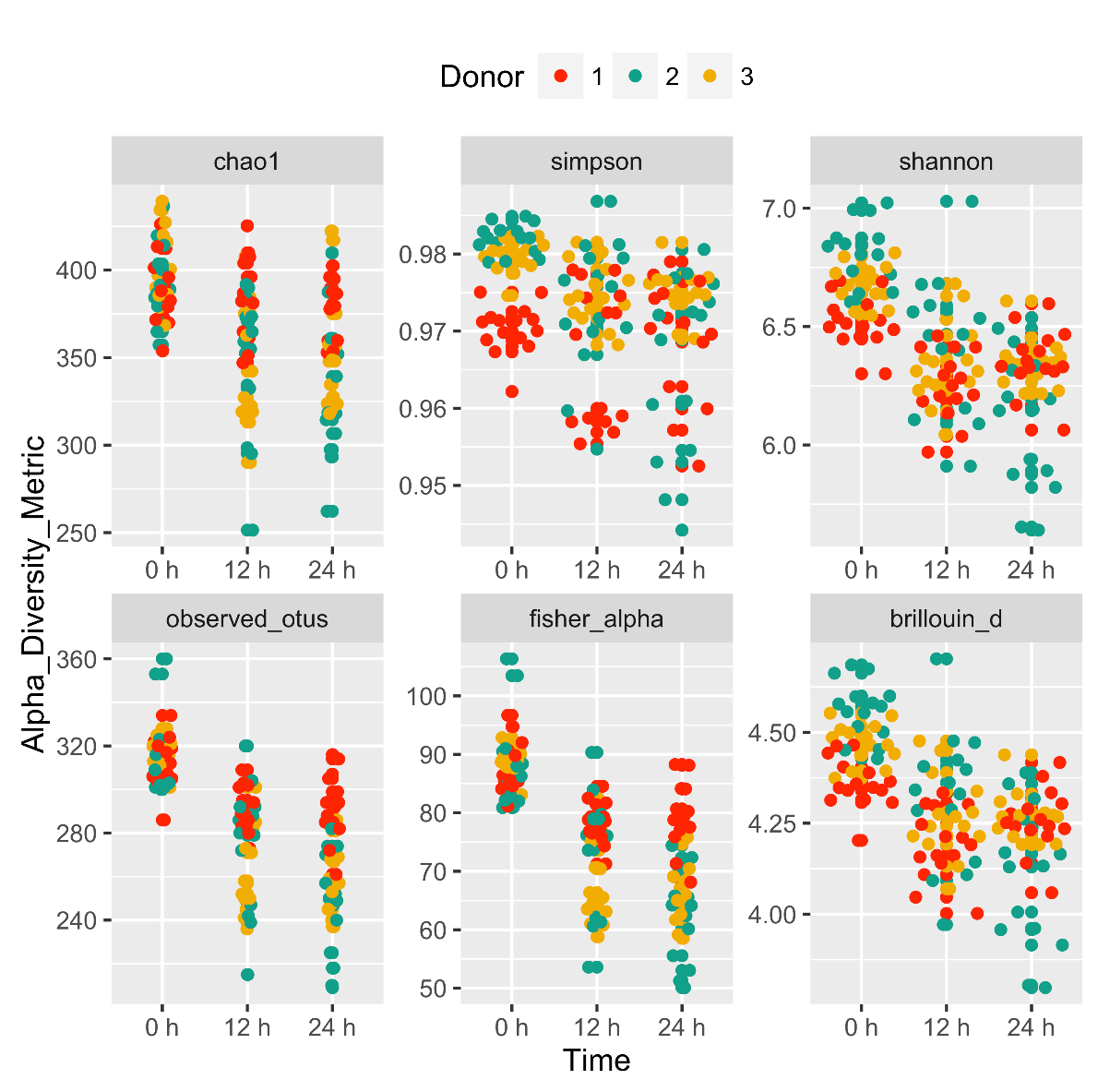


Figure S5. Six metrics of analysis for alpha-diversity among samples at 0, 12 and 24 h of analysis, grouped by treatment for all three fecal donors.


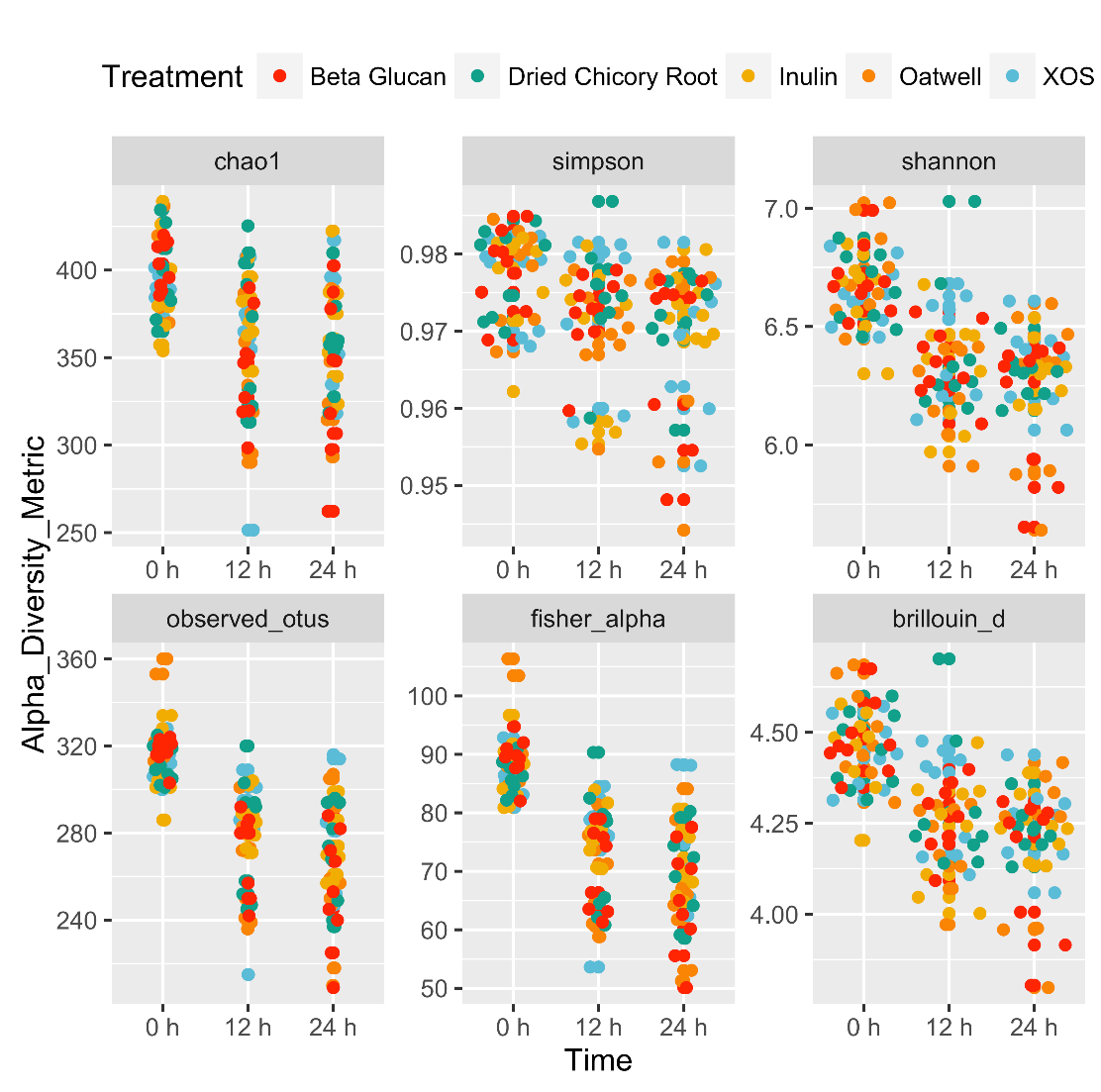


Figure S6. Bray-Curtis β-diversity principal component analysis of technical replicates among each treatment group between microbiota analysis of three fecal donors.


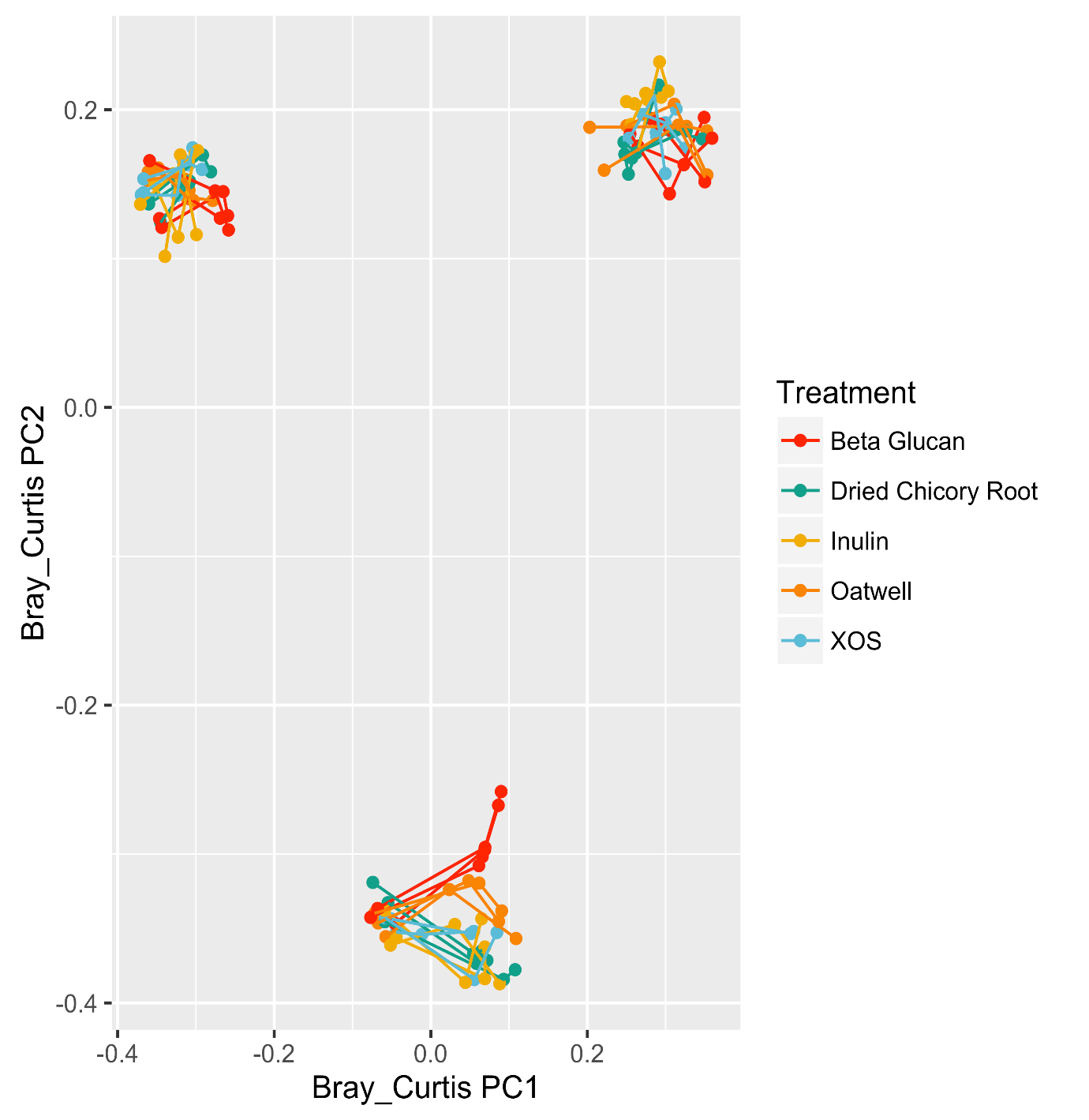


Donor 1

Donor 3

Donor 2

Figure S7. Bray-Curtis β-diversity principal component analysis among microbiota of three fecal donors at 0, 12 and 24 h of analysis.


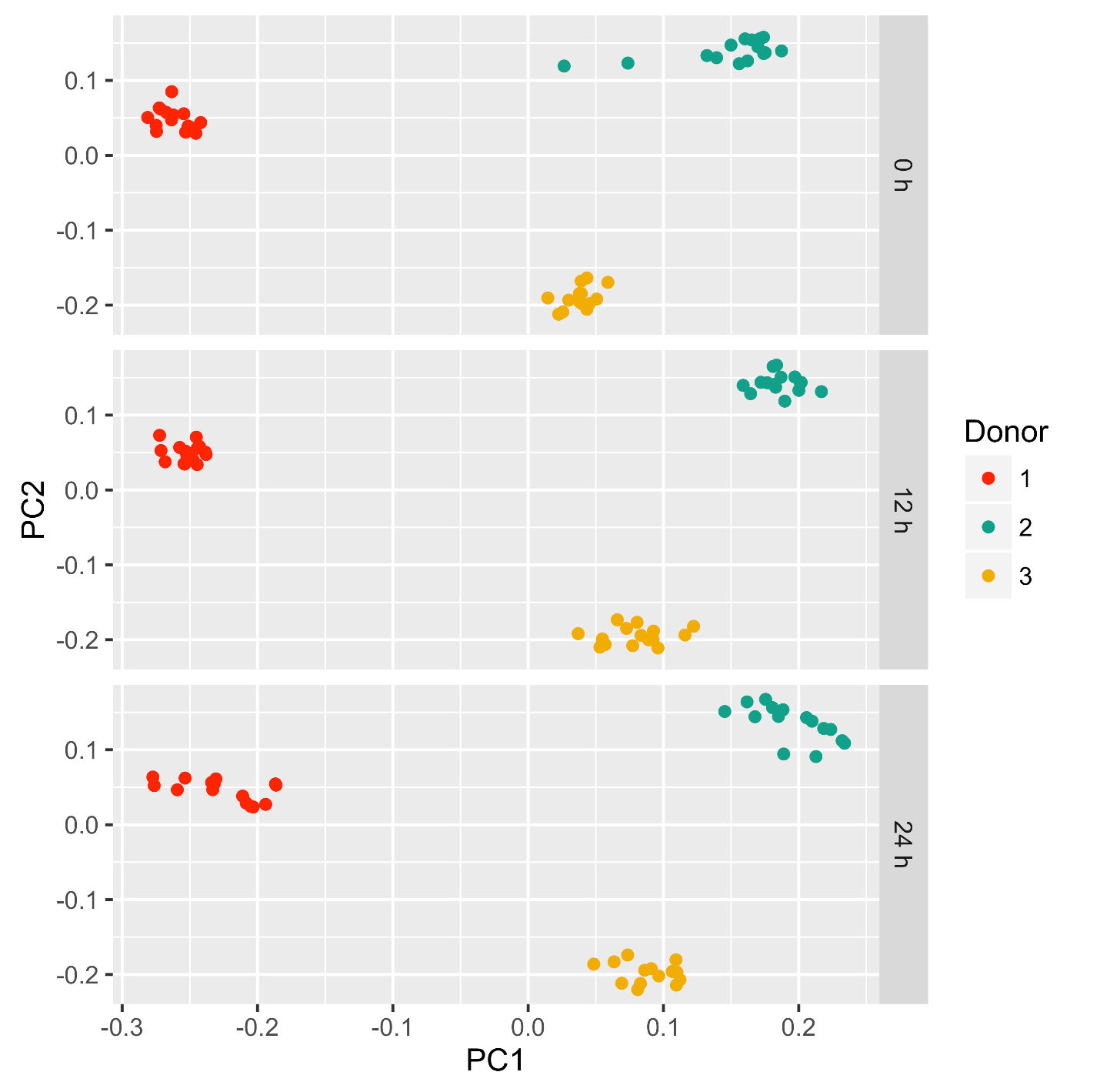


Figure S8. Variations in abundant phyla among three donors analyzed.


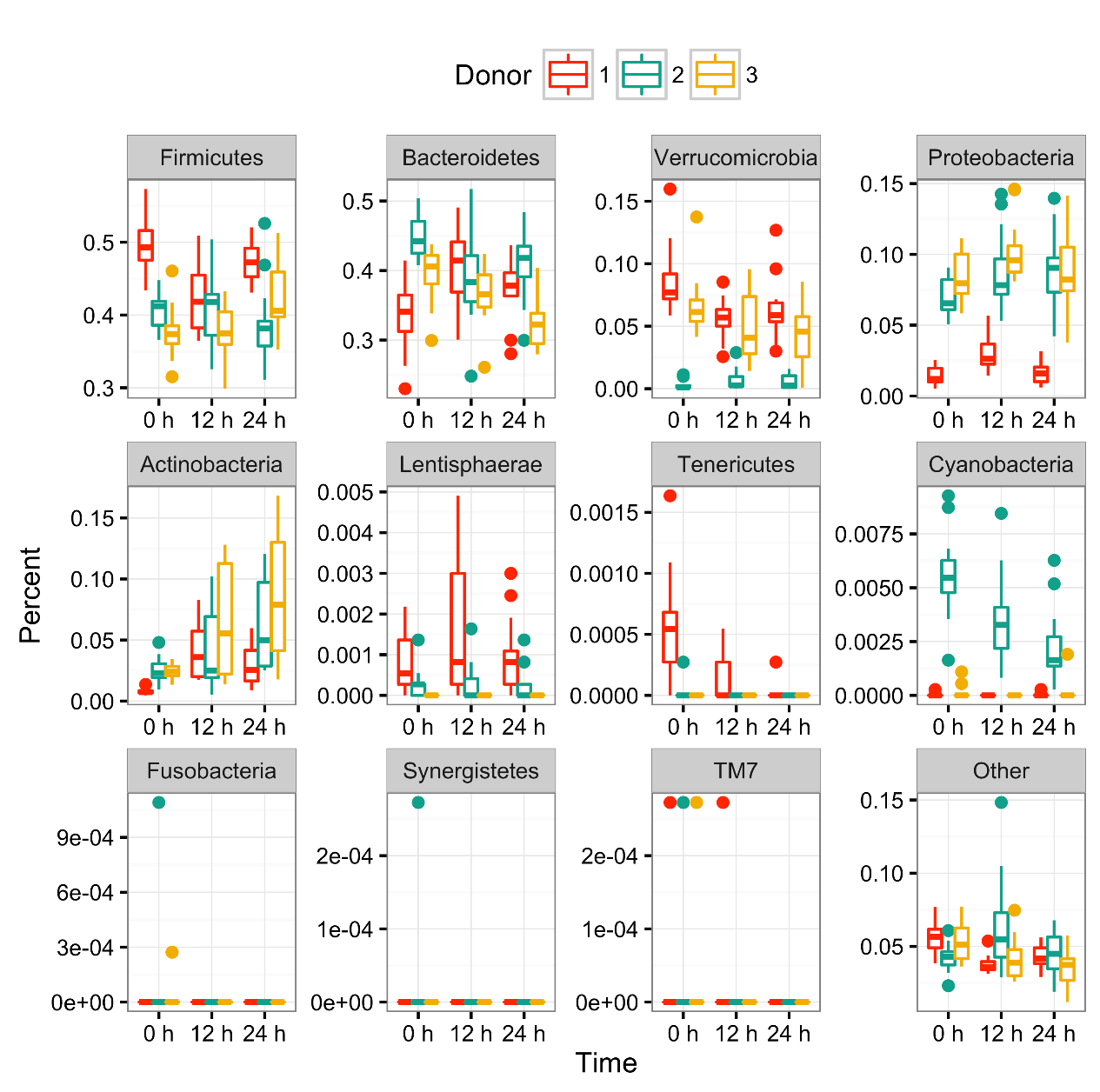


Figure S9. Variations in treatment groups and pooled donors analyzed.


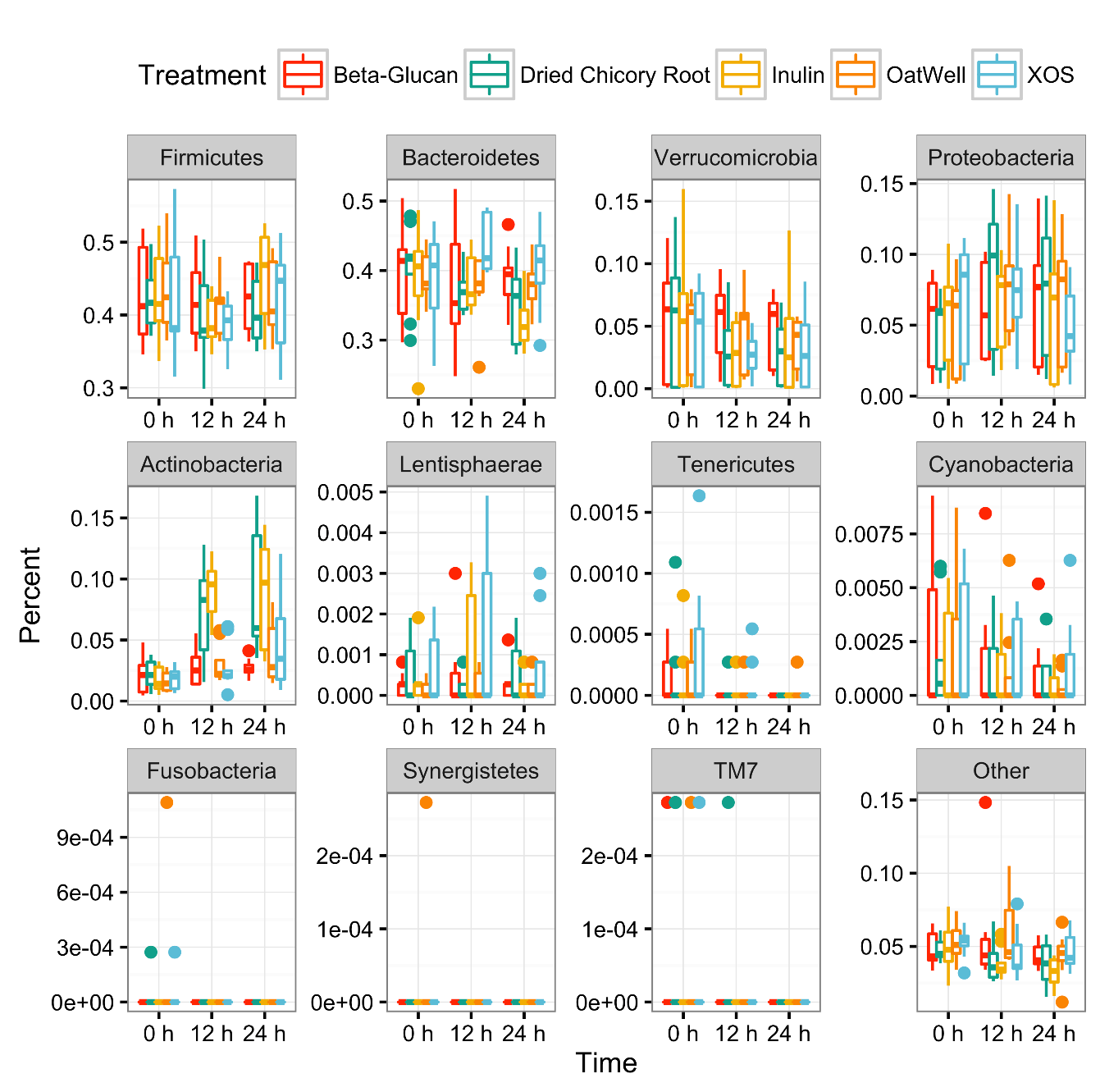

Supplement: Supplementary file 1 [file nutrients-09-01361-s001.docx]
